# 

**Published:** 1919

**Authors:** 


CONTENTS. *Page*

The Systematic Examination of the Abdomen. By R. G. P. Lansdown, M.B., B.S. Durh. ... 33

Postgraduate Study. By J. M. Fortescue-Brickdale, M.A., M.D., M.R.C.P. ... 48

The Operative Findings in Thirty Cases of Gunshot Injury of Nerves, with Remarks on Diagnosis, Localisation, and the Technique of Operation. By C. A. Morton, F.R.C.S. ... 55

Reviews of Books ... 81

Editorial Notes ... 87

The Library of the Bristol Medico-Chirurgical Society ... 93

Meetings of Societies ... 100

Obituary Notice:

Barclay Josiah Baron, Knight ... 100

Local Medical Notes ... 103

